# Riboflavin as a Mucosal Adjuvant for Nasal Influenza Vaccine

**DOI:** 10.3390/vaccines9111296

**Published:** 2021-11-09

**Authors:** Yinyan Yin, Jinyuan Wang, Xing Xu, Bangyue Zhou, Sujuan Chen, Tao Qin, Daxin Peng

**Affiliations:** 1 College of Medicine, Yangzhou University, Yangzhou 225009, China; yyyin@yzu.edu.cn (Y.Y.); MX120201034@yzu.edu.cn (B.Z.); 2 College of Veterinary Medicine, Yangzhou University, Yangzhou 225009, China; MX120190761@yzu.edu.cn (J.W.); 182002128@yzu.edu.cn (X.X.); chensj@yzu.edu.cn (S.C.); qintao@yzu.edu.cn (T.Q.); 3 Jiangsu Co-Innovation Center for the Prevention and Control of Important Animal Infectious Disease and Zoonoses, Yangzhou University, Yangzhou 225009, China; 4 Joint International Research Laboratory of Agriculture and Agri-Product Safety, The Ministry of Education of China, Yangzhou University, Yangzhou 225009, China; 5 Jiangsu Research Centre of Engineering and Technology for Prevention and Control of Poultry Disease, Yangzhou University, Yangzhou 225009, China; 6 Jiangsu Key Laboratory of Experimental and Translational Non-Coding RNA Research, Yangzhou University, Yangzhou 225009, China

**Keywords:** adjuvant, influenza nasal vaccine, riboflavin, dendritic cells, MAPK pathways

## Abstract

Intranasal immunization with whole inactivated virus (WIV) is an important strategy used for influenza prevention and control. However, a powerful mucosal adjuvant is required to improve nasal vaccine efficacy. Riboflavin, as a food additive with the advantages of being safe and low-cost, widely exists in living organisms. In this paper, the mucosal adjuvant function of riboflavin was studied. After intranasal immunization with H1N1 WIV plus riboflavin in mice, we found that the mucosal immunity based on the secretory IgA (sIgA) levels in the nasal cavity, trachea, and lung were strongly enhanced compared with H1N1 WIV alone. Meanwhile, the IgG, IgG1, and IgG2a levels in serum also showed a high upregulation and a decreased ratio of IgG1/IgG2a, which implied a bias in the cellular immune response. Moreover, riboflavin strongly improved the protection level of H1N1 inactivated vaccine from a lethal influenza challenge. Furthermore, riboflavin was found to possess the capacity to induce dendritic cell (DC) phenotypic (MHCII, CD40, CD80, and CD86) and functional maturation, including cytokine secretion (TNF-α, IL-1β, IL-12p70, and IL-10) and the proliferation of allogeneic T cells. Lastly, we found that the DC maturation induced by riboflavin was dependent on the activation of the mitogen-activated protein kinase (MAPK) signaling pathway, which plays an important role in immune regulation. Therefore, riboflavin is expected to be developed as an alternative mucosal adjuvant for influenza nasal vaccine application.

## 1. Introduction

Influenza is a highly infectious zoonosis that can cause human and animal death [1]. In 1918, a deadly influenza pandemic caused by the H1N1 influenza virus infected approximately 500 million people and resulted in 50 to 100 million people dying worldwide [2]. In 2009, a new H1N1 subtype influenza virus spread rapidly worldwide among humans and caused approximately 18,500 worldwide deaths for the period of April 2009 to August 2010 [3]. Vaccination is currently still an effective strategy for the prevention and control of influenza. It is known that the nasal cavity is the primary entry site of influenza virus. Therefore, if mucosal immune protection is established in the nasal mucosa, early viral infection and shedding will be cut off due to the secretory IgA (sIgA) antibody at the mucosal site [4,5]. Inactivated influenza vaccines with a good level of safety are widely used. However, the development of inactivated vaccines based on the intranasal route is limited due to the existence of mucosal barriers [6], which impede the delivery of antigens to submucosal antigen-presenting cells (APCs)—dendritic cells (DCs)—and then restrict the subsequent DC maturation levels to initiate protective adaptive immunity [5]. It is of note that the development of mucosal adjuvants is an important strategy that is used to enhance the immune efficacy of inactivated nasal vaccines. Cholera toxin (CT) is a classic adjuvant of the mucosal immune system [7], but toxicity restricts its application. Previously, we proposed a new strategy to mobilize broad submucosal DCs for the transepithelial sampling of influenza WIV and subsequent DC maturation using CpG oligodeoxynucleotides [8]. However, the price of synthesis limits their wide application. Therefore, mucosal adjuvants that are highly efficient and safe and that have a DC-targeting function will be developed in the future [5].

**Figure 1:**
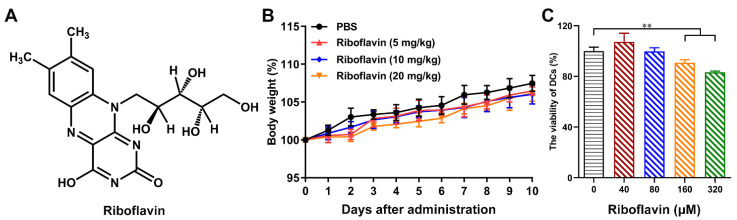
Biosafety of riboflavin in vivo and in vitro. (**A**) Chemical structure of riboflavin. (**B**) Riboflavin at different concentrations (5, 10, and 20 mg/kg body weight) was intranasally administered to mice. The body weight of the mice was monitored continuously for 10 days (*n* = 10/group). (**C**) The cytotoxicity of riboflavin (40 μM, 80 μM, 160 μM, and 320 μM) in DCs was detected by the CCK-8 assay. All of the data are presented as the mean ± SD. Statistical significance was assessed by one-way ANOVA to compare the results between different groups; ** *p* < 0.01.

Riboflavin (Figure 1A), also referred to as vitamin B2, is a water-soluble member of the B vitamin family [9]. Its history can be traced back to 1879 when it was isolated from milk. Due to its isoalloxazine ring (*flavus* from latin for yellow) and *ribo* from the ribito chain, it was further renamed riboflavin due to its chemical structure. The vitamin properties of this yellow pigment were identified in early 1930, revealing the role of its bioactive forms, flavin mononucleotide (FMN) and flavin adenine dinucleotide (FAD) [10,11]. The derivatives FAD and FMN play vital roles as cofactors for enzyme-catalyzed reactions, and some studies have considered the notion that FAD and FMN are important cofactors in the production of adenosine triphosphate (ATP), which is closely related to cellular immune responses [12]. Furthermore, riboflavin is used in dietary supplements and in the treatment of inflammatory diseases, such as glossitis, cheilitis, sepsis, cataracts, and migraines [9]. Moreover, some reports have found that riboflavin can enhance the phagocytic activity of neutrophils and macrophages and stimulate the multiplication of neutrophils and monocytes [9]. Riboflavin is a safe food additive that has been approved by the Food and Drug Administration (FDA). It is present in large amounts in many food items, such as meat, eggs, vegetables, and seafood [13,14]. Furthermore, *B. subtilis* and *Ashbya gossypii* have been shown to produce riboflavin in large quantities [15,16]. Therefore, riboflavin has potential as a vaccine adjuvant considering its safety, price, and immune function.

Here, an influenza nasal vaccine was prepared by combination with H1N1 WIV and riboflavin, and both the immune efficacy and challenge protection levels were evaluated in mice. Furthermore, we clarified the potential mechanism from the perspective of DC maturation and the possible MAPK signaling pathway.

## 2. Materials and Methods

### 2.1. Ethics Statements

The Jiangsu Administrative Committee for Laboratory Animals (permission number: SYXK(SU)2017-0044) approved the animal studies conducted for this paper according to the guidelines for laboratory animal welfare and ethics of the Jiangsu Administrative Committee for Laboratory Animals.

### 2.2. Experimental Animals and Materials

Four to six week old BALB/c and C57BL/6 mice were obtained from the Animal Research Center of Yangzhou University (Yangzhou, China). The mice were acclimatized for at least 7 days before their use under specific pathogen-free (SPF) conditions.

Riboflavin was sourced from Sangon Biotech (Shanghai, China). Cholera toxin B subunit (CTB) was from Sigma-Aldrich (St. Louis, MO, USA). Recombinant mouse interleukin-4 (IL-4) and granulocyte-macrophage colony-stimulating factor (GM-CSF) were sourced from Peprotech (Rocky Hill, NJ, USA). FR 180204 (ERK inhibitor), BIRB 796 (P38 MAPK inhibitor), SP600125 (JNK inhibitor), and the CCK-8 kit were obtained from Beyotime (Shanghai, China). Flow cytometry (FCM) antibodies, including fluorescein isothiocyanate (FITC)–CD4, phycoerythrin (PE)–CD8, allophycocyanin (APC)–CD3, FITC–CD86, FITC–MHCII, PE–CD80, PE–CD40, APC–CD69, PE/Cy7–CCR7, and APC/Cy7–CD4 or their respective isotype controls were sourced from BD Bioscience (Franklin Lakes, NJ, USA). Western blot antibodies from rabbits, including anti-Erk1 (pT202/pY204) + Erk2 (pT185/pY187) antibody (p-ERK), anti-ERK1/2 antibody (ERK), anti-P38 (T180 + Y182) antibody (p-P38), anti-P38 antibody (P38), anti-JNK antibody (JNK), and anti-phospho-JNK1/2/3(T183 + T183 + T221) antibody (p-JNK) were obtained from Huabio (Hangzhou, China). Goat anti-rabbit IgG–horseradish peroxidase (HRP) was from Bioworld (St. Louis Park, MN, USA). Mouse anti-β-actin was obtained from TransGen Biotech (Beijing, China). Goat anti-mouse IgG–HRP was obtained from abcam (Cambridge, UK). HRP-conjugated anti-mouse IgG, IgG1, and IgG2a were obtained from Santa Cruz Biotechnology (Dallas, TX, USA). HRP-conjugated anti-mouse IgA was sourced from Southern Biotech (Birmingham, AL, USA). HiScript II Q Select RT SuperMix for qPCR and AceQ qPCR SYBR Green Master Mix were sourced from Vazyme (Nanjing, China). The primer for NP was sourced from TsingKe Biotech (Beijing, China). Fetal bovine serum (FBS) and RPMI 1640 medium were obtained from Thermo Fisher Scientific (Waltham, MA, USA). Penicillin and streptomycin were sourced from Invitrogen (Grand Island, NY, USA). Carboxyfluorescein succinimidyl ester (CFSE) was sourced from Invitrogen (Grand Island, NY, USA).

### 2.3. Preparation of H1N1 WIV

The influenza viruses A/Puerto Rico/8/34 (H1N1) were stored in our laboratory and further purified via sucrose density gradient centrifugation using our improved method [5]. The influenza viruses were inactivated at 56 °C for 0.5 h and inoculated in 10 day old SPF embryonated eggs for three passages to detect the complete loss of infectivity. Additionally, the protein concentration of inactivated influenza viruses was measured by the bicinchoninic acid (BCA) protein assay kit (Thermo Fisher Scientific, Waltham, MA, USA).

### 2.4. Immunogenicity Study

A total of 45 6 week old BALB/c mice were purchased, 40 of which were chosen for future study according to inclusion criteria based on equal mean body weight and healthy status. Mice (*n* = 10/group) were immunized intranasally with H1N1 WIV (1.75 µg hemagglutinin (HA)) alone or in combination with riboflavin (100 µg) or CTB (10 µg) two times at 0 and 14 days. Twenty-eight days after the primary immunization, the mice were euthanized. Nasal, tracheal, and lung lavage fluid samples were washed with 0.5, 0.2, and 0.5 mL of sterile PBS, respectively. Serum hemagglutination inhibition (HI) titers of the immunized mice were analyzed according to a previously described procedure [17]. Antigen-specific sIgA antibodies in mucosal washes (nasal, tracheal, and lung wash) and IgG (total IgG, IgG1, and IgG2a) in serum were detected by enzyme-linked immunosorbent assay (ELISA) as described previously [18]. Furthermore, splenic lymphocytes were isolated and analyzed to determine the percentages of CD3^+^ CD4^+^ and CD3^+^ CD8^+^ splenic T cells via FCM.

### 2.5. Virus Challenge

Two weeks after the final immunization, mice were anesthetized and intranasally challenged with a 10^6^ egg infectious dose (EID_50_) of H1N1 influenza virus. The survival rates and body weight changes of challenged mice (*n* = 10/group) were recorded for 14 days. At 5 days post infection (p.i.), lung tissues were collected for the histopathological examination or virus load. Microscopic lesions were assessed by two blinded veterinary pathologists. The scoring of lesions was conducted according to scales adapted from the work of Gauger et al. [19]. Each lung tissue was ground in 0.4 mL of sterile PBS containing antibiotics and RNase inhibitor, and then the total RNA was extracted. The NP gene was detected by quantitative reverse transcription PCR (qRT-PCR) and housekeeping gene β-actin was used as an internal standard. The primer sequences were as follows: NP (F: GGGCAGAACGTCTGACATGA, R: GGGTTCGTTGCCTTTTCGTC) and β-actin (F: CATCCGTAAAGACCTCTATGCCAAC, R: ATGGAGCCACCGATCCACA).

### 2.6. Isolation and Culture of DCs

DCs were cultured using our advanced method [20]. In short, bone marrow was extracted from the tibias and femurs of wild-type C57BL/6 mice and incubated with red blood cell lysing buffer (Beyotime, Shanghai, China). Then, the cells were cultured in complete medium (RPMI 1640 with 10% FBS, 10 ng/mL IL-4 and GM-CSF, 1% penicillin/streptomycin). After about 60 h of culture, nonadherent granulocytes were discarded, and fresh culture medium was added. After 6 days, nonadherent and loosely adherent cells were collected and subcultured overnight. On day 7, >90% of CD11c^+^ cells were used.

### 2.7. Phenotype and Migration Assay of DCs

In vitro, the DCs were treated with riboflavin (40 µM) for 24 h. Next, the cells were collected and washed with sterile PBS and stained with PE–CD40, FITC–MHCII, FITC–CD86, PE–CD80, PE/Cy7–CCR7, or their respective isotypes for 30 min at 4 °C. After this, the cells were analyzed via FCM.

### 2.8. Cytokine Assay of DCs

The secretion of TNF-α, IL-1β, IL-12p70, and IL-10 in culture supernatants was detected by ELISA kits (eBioscience, San Diego, CA, USA) according to the manufacturer’s instructions.

### 2.9. Allogenic Mixed Lymphocyte Reaction (MLR) Assay

Responder T cells for the MLR assay were isolated from BALB/c mice splenic lymphocytes using an isolation kit of CD4^+^ T cells (Miltenyi Biotech, Gladbach, Germany). Next, the CD4^+^ T cells were labeled with CFSE (Invitrogen, Carlsbad, CA, USA) according to the manufacturer’s instructions and cocultured for 5 days in duplicate with DCs (DC:T cell ratios of 1:1 and 1:5). The proliferation of CD4^+^ T cells was detected via FCM.

### 2.10. Western Blot

The DCs were stimulated with riboflavin (40 µM) for 1 h. Next, the cells were washed with ice-cold sterile PBS and lysed with 200 µL of RIPA buffer (Beyotime, Shanghai, China) containing phosphatase and protease inhibitors. The lysates were centrifuged for 10 min at 12,000× *g*, and then the protein concentrations were measured using the BCA protein assay kit. The protein was separated on SDS-polyacrylamide gels and then transferred to the polyvinylidene fluoride membrane. Next, the membrane was blocked with 5% skim milk for 2 h and incubated with rabbit anti-p-ERK, anti-ERK, anti-p-P38, anti-P38, anti-p-JNK, and anti-JNK antibodies or mouse anti-β-actin antibody at 4 °C overnight. After being washed with TBST, the membrane was immunolabeled with goat anti-rabbit IgG-HRP or goat anti-mouse IgG-HRP for 1 h at room temperature, followed by an enhanced chemiluminescence reagent (Pierce, Rockford, IL, USA) to visualize the protein of the membrane. Autoradiograms were scanned and analyzed using Quantity One (Bio-Rad, Hercules, CA, USA) to quantify the band densities.

### 2.11. Statistical Analysis

Results were expressed as the means ± standard deviations (SD). Unpaired Student’s two-sided *t*-tests were used to determine the statistical significance between the two groups. A one-way ANOVA analysis of variance was employed to compare the variance between the different groups; * *p* < 0.05, ** *p* < 0.01.

## 3. Results

### 3.1. Riboflavin Enabled Inactivated Influenza Vaccine to Trigger the Local Mucosal and Systemic Immune Responses

Firstly, we found that riboflavin (10–80 μM) significantly stimulated the proliferation of splenic lymphocytes compared with the control, implying that riboflavin has the ability to activate the host immune response and might be developed as a potential vaccine adjuvant (Figure S1, Supplementary Materials). In addition, the biosafety also should not be overlooked when riboflavin is used in vivo. As shown in Figure 1B, there was no change in murine body weight in the riboflavin group via intranasal dropping compared with the PBS group, even though the dose was up to 20 mg/kg. In vitro, we also did not find cytotoxicity of riboflavin (≤80 μM) in DCs (Figure 1C), which act as APCs to trigger a downstream series of immune responses [21]. These data indicate that riboflavin has good biosafety.

**Figure 2:**
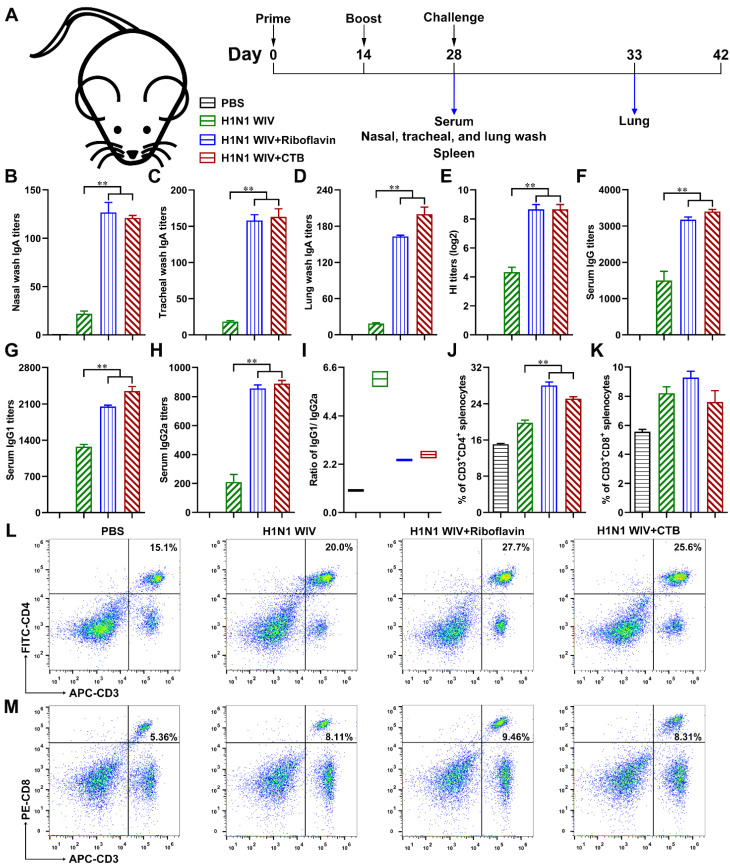
H1N1 WIV-specific local mucosal and systemic immunity after the intranasal immunization of mice. (**A**) Scheme of immunization (*n* = 10/group), challenge, and sampling. (**B**–**D**) Twenty-eight days after the primary immunization, antigen-specific mucosa sIgA titers in the nasal wash (**B**), tracheal wash (**C**), and lung wash (**D**) of immunized mice were measured by ELISA. (**E**) Serum hemagglutination inhibition (HI) titers of immunized mice were measured by the HI assay. (**F**–**H**) Antigen-specific serum IgG titers (**F**), IgG1 titers (**G**), and IgG2a titers (**H**) of immunized mice were measured by ELISA. (**I**) The ratio of IgG1/IgG2a. (**J**–**M**) The percentages of CD3^+^ CD4^+^ (**J**,**L**) and CD3^+^ CD8^+^ (**K**,**M**) splenic T cells of immunized mice were analyzed via FCM. All of the data are presented as the mean ± SD. Statistical significance was assessed by one-way ANOVA to compare the results between different groups; ** *p* < 0.01.

Furthermore, to study whether riboflavin might function as a nasal mucosal adjuvant for H1N1 WIV, the mice were immunized two times intranasally with H1N1 WIV alone, H1N1 WIV plus riboflavin, or H1N1 WIV in combination with an effective mucosal adjuvant CTB (Figure 2A). Fourteen days after the final immunization, the antigen-specific sIgA levels in the respiratory mucosa were detected by ELISA in order to evaluate the mucosal immunity. The results showed that the sIgA antibodies in the nasal (Figure 2B), tracheal (Figure 2C), and lung (Figure 2D) wash samples induced by riboflavin/CTB plus H1N1 WIV were significantly greater than those induced by H1N1 WIV alone. Moreover, blood samples were also collected from the mice of each group, and HI titers were measured by HI assay in order to evaluate the systemic immune response. We found that the HI titers were markedly increased in the H1N1 WIV plus riboflavin group compared with those in the H1N1 WIV alone group (Figure 2E). Furthermore, the antigen-specific serum IgG and its subtypes were also measured by ELISA. As shown in Figure 2F,G,H, respectively, the IgG, IgG1, and IgG2a levels in the H1N1 WIV plus riboflavin/CTB group were substantially higher than those in the H1N1 WIV alone group. It is known that the IgG1 response reflects the activity of T helper cell type 2 (Th2) CD4^+^ T cells, while the IgG2a response results from T helper cell type 1 (Th1) activity [22]. Our results showed that the IgG1/IgG2a ratio was >1 in the H1N1 WIV alone group or H1N1 WIV plus riboflavin/CTB group (Figure 2I), suggesting that H1N1 WIV alone or in combination with riboflavin/CTB induced a biased Th2-type antibody response. Meanwhile, H1N1 WIV plus riboflavin/CTB was found to induce a much higher percentage of CD3^+^ CD4^+^ splenic T cells than H1N1 WIV alone (Figure 2J–M). Altogether, these results indicate that riboflavin strongly induces mucosal and systemic immune responses in vivo and could be a potent mucosal adjuvant for inactivated influenza vaccines by intranasal immunization.

### 3.2. Riboflavin Strongly Improved the Protection Levels of Influenza Vaccine against H1N1 Influenza Virus Attack

**Figure 3:**
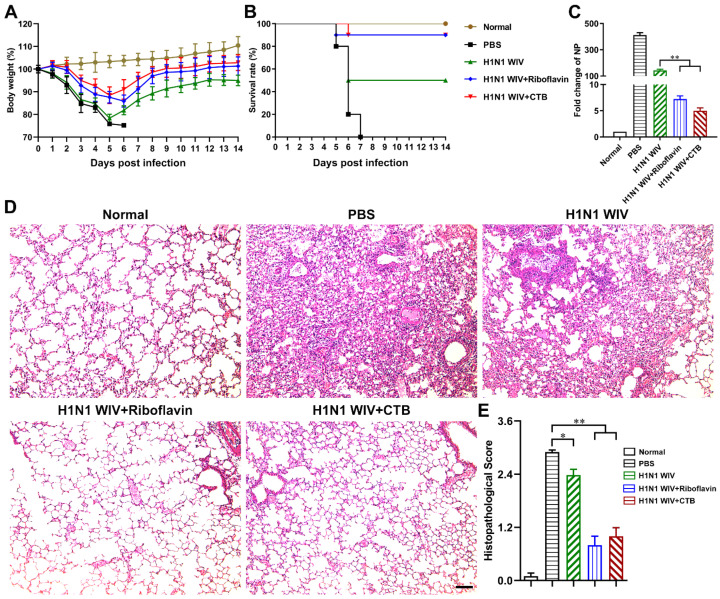
Intranasal vaccination with H1N1 WIV plus riboflavin protects mice from H1N1 influenza virus attack. Twenty-eight days after the primary immunization, mice were anesthetized and intranasally challenged with 10^6^ EID_50_ of H1N1 influenza virus. (**A**,**B**) The average body weights (*n* = 10/group) and survival rates (*n* = 10/group) in each group were monitored daily for 14 days. On day 5 p.i., lung tissues were collected. (**C**) The levels of NP gene in lung tissues (*n* = 5/group) were detected by qRT-PCR. (**D**) Representative histopathological changes in H&E (hematoxylin and eosin)-stained lung tissues (*n* = 3/group). Bars: 500 μm. (**E**) Histopathologic scores of lungs. All of the data are presented as the mean ± SD. Statistical significance was assessed by one-way ANOVA to compare the results between different groups; * *p* < 0.05, ** *p* < 0.01.

To verify the protective efficacy of intranasal vaccination with H1N1 WIV plus riboflavin, the immunized mice were intranasally challenged with 10^6^ EID_50_ of H1N1 influenza virus (A/Puerto Rico/8/34) 28 days after the primary immunization. During the 14 day observation period, we found that the mice immunized with H1N1 WIV plus riboflavin recovered after a slight loss of body weight (Figure 3A), and their survival rate of 90% was same as that of the CTB group (Figure 3B). However, the mice in the H1N1 WIV alone group showed severe weight loss (Figure 3A) and their survival rate remained at 50% until the end of the 2 week infection (Figure 3B). Furthermore, the NP gene levels of H1N1 influenza virus in murine lungs were detected by qRT-PCR in order to evaluate the viral load. By 5 days p.i., the fold change of the NP gene in the H1N1 WIV plus riboflavin/CTB group was remarkably lower than that in the H1N1 WIV alone group (Figure 3C). Moreover, the pathological status of murine lung tissues was examined using an H&E staining method, which showed that the lung tissues from the PBS group exhibited severe pneumonia with inflammatory cellular infiltration, pneumonorrhagia, alveolar wall edema, and thickening on day 5 p.i. (Figure 3D,E). However, the lung tissues from the H1N1 WIV plus riboflavin/CTB group showed a slight histopathological change (Figure 3D,E). Accordingly, these observations indicate that riboflavin as a mucosal adjuvant significantly improves the protection level of the nasal influenza vaccine against H1N1 virus attack in mice.

### 3.3. Riboflavin Promoted the Phenotypic Maturation of DCs

**Figure 4:**
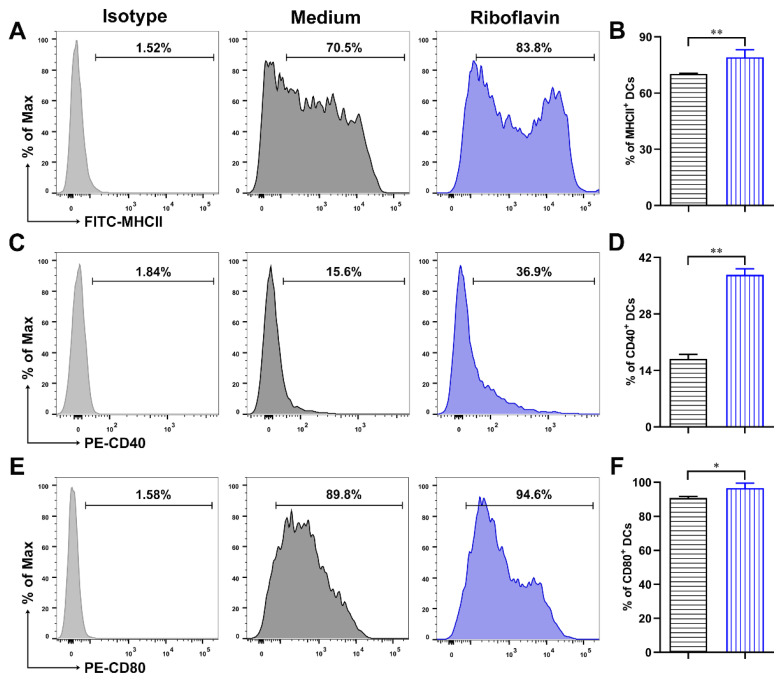

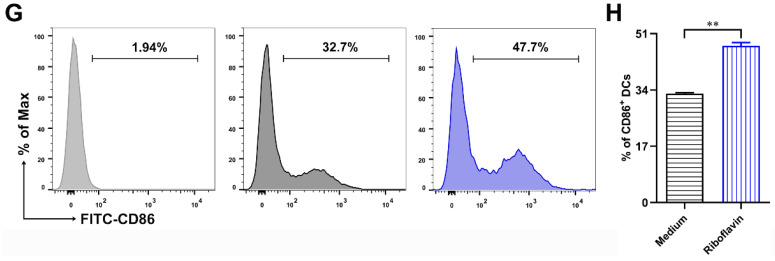
Riboflavin promotes the expression of MHCII, CD40, CD80, and CD86 in DCs. DCs were stimulated with riboflavin (40 µM) for 24 h. The expression of MHCII (**A**), CD40 (**C**), CD80 (**E**), and CD86 (**G**) was detected via FCM. (**B**,**D**,**F**,**H**) The percentage values are presented as the mean ± SD. Results are from one representative experiment out of three performed. Unpaired Student’s two-sided *t*-tests were employed to determine the differences between the two groups; * *p* < 0.05, ** *p* < 0.01.

Next, to investigate whether riboflavin could activate DCs to enhance the downstream immune response, the expressions of phenotypic molecules were detected via FCM. After stimulation for 24 h with riboflavin, the expressions of MHCII (Figure 4A,B), CD40 (Figure 4C,D), CD80 (Figure 4E,F), and CD86 (Figure 4G,H) on DCs were significantly upregulated compared with those of the control. These results indicate that riboflavin improves the phenotypic maturation of DCs.

### 3.4. Riboflavin Enhanced the Cytokine Production of DCs

**Figure 5:**
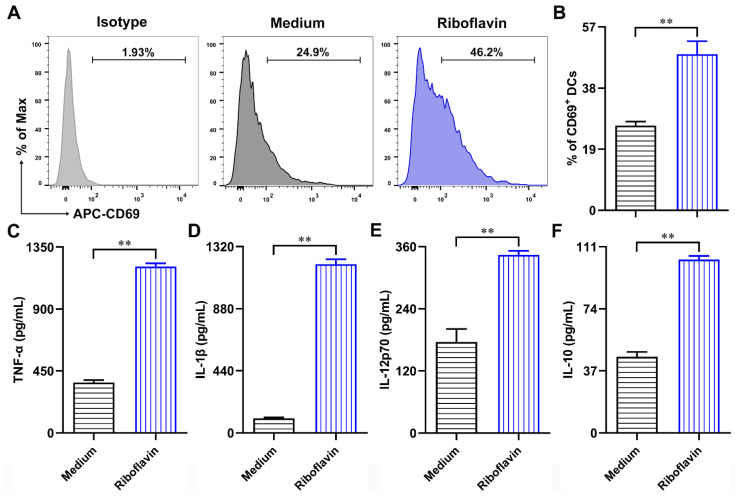
Riboflavin enhances the secretion of cytokines in DCs. DCs were incubated with riboflavin (40 µM) for 24 h. (**A**,**B**) The expression of activation marker CD69 was detected via FCM. (**C**–**F**) Supernatants were collected and detected for TNF-α, IL-1β, IL-12p70, and IL-10 by ELISA. Results are from one representative experiment out of three performed. All of the data are presented as the mean ± SD. Unpaired Student’s two-sided *t*-tests were employed to determine the differences between the two groups; ** *p* < 0.01.

Next, CD69 was detected as a critical activation marker of DCs [23]. The results showed that the expression of CD69 in DCs was remarkably increased after treatment with riboflavin for 24 h (Figure 5A,B). Furthermore, the levels of cytokines in supernatants were also detected by ELISA. After incubation with riboflavin for 24 h, the production of TNF-α (Figure 5C), IL-1β (Figure 5D), IL-12p70 (Figure 5E), and IL-10 (Figure 5F) in DC supernatants was appreciably increased. Taken together, these data indicate that riboflavin enhances the cytokine production of DCs.

### 3.5. Riboflavin Improved the Migration Marker CCR7 Expression of DCs

**Figure 6:**
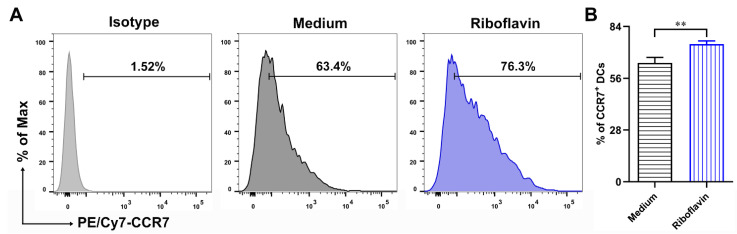
Riboflavin enhances the expression of CCR7 in DCs. DCs were incubated with riboflavin (40 µM) for 24 h. (**A**) The expression of migration marker CCR7 was detected via FCM. (**B**) The percentage values are presented as the mean ± SD. Results are from one representative experiment out of three performed. Unpaired Student’s two-sided *t*-tests were employed to determine the differences between the two groups; ** *p* < 0.01.

To demonstrate whether riboflavin might modulate the expression of migration marker CCR7 in DCs, the cells were incubated with riboflavin for 24 h. Additionally, the expression levels of CCR7 in DCs were analyzed via FCM. As shown in Figure 6A,B, riboflavin significantly upregulated the expression of migration marker CCR7 in DCs. These findings suggest that riboflavin improves the expression of migration marker CCR7 in DCs.

### 3.6. Riboflavin-Induced DCs Improved the Allostimulatory Capacity on CD4^+^ T Cells

**Figure 7:**
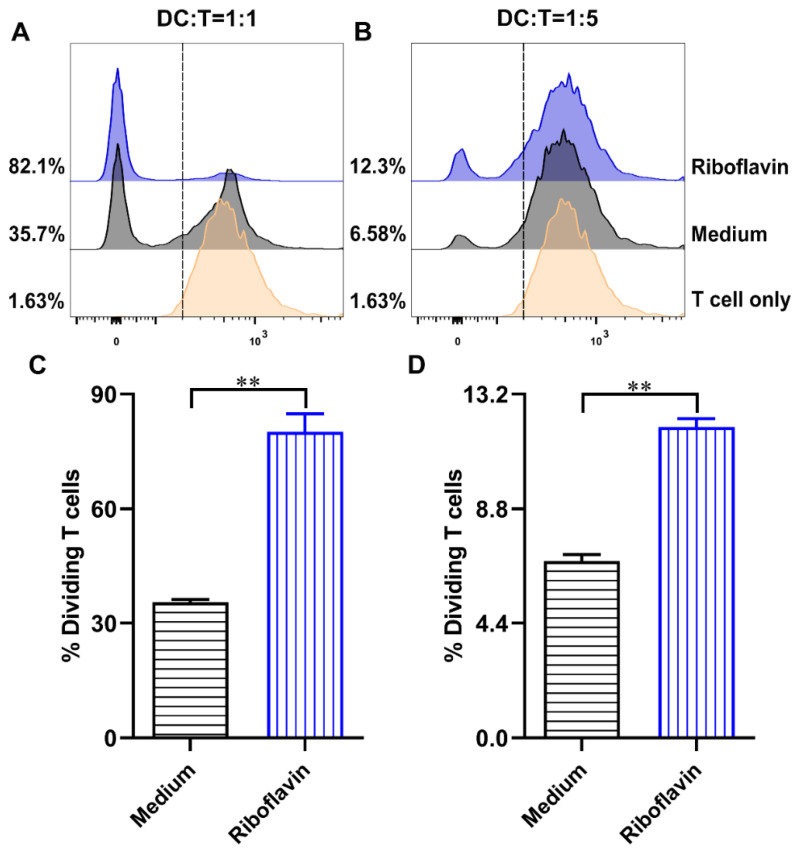
Riboflavin-induced DCs promote the proliferation of allogeneic CD4^+^ T cells. DCs were incubated with riboflavin (40 µM) for 24 h and then cocultured with CFSE-labeled CD4^+^ T cells in two graded cell numbers (DC/T-cell ratios: 1:1 (**A**) and 1:5 (**B**)). After 5 days, the proliferation of CD4^+^ T cells was detected via FCM. (**C**,**D**) The percentage values are presented as the mean ± SD. Results are from one representative experiment out of three performed. Unpaired Student’s two-sided *t*-tests were employed to determine the differences between the two groups; ** *p* < 0.01.

To investigate whether DCs might act as a fully functional APC, the treatment of DCs with riboflavin for 24 h was evaluated to determine their ability to stimulate allogeneic CD4^+^ T cells. As shown in Figure 7, the riboflavin-induced DCs stimulated proliferative responses more effectively than untreated DCs at all ratios of DC:T cell tests. These results suggest that the riboflavin-induced DCs strongly improve the allostimulatory capacity in CD4^+^ T cells.

### 3.7. Riboflavin Promoted DC Maturation via Activation of the MAPK Pathway

**Figure 8:**
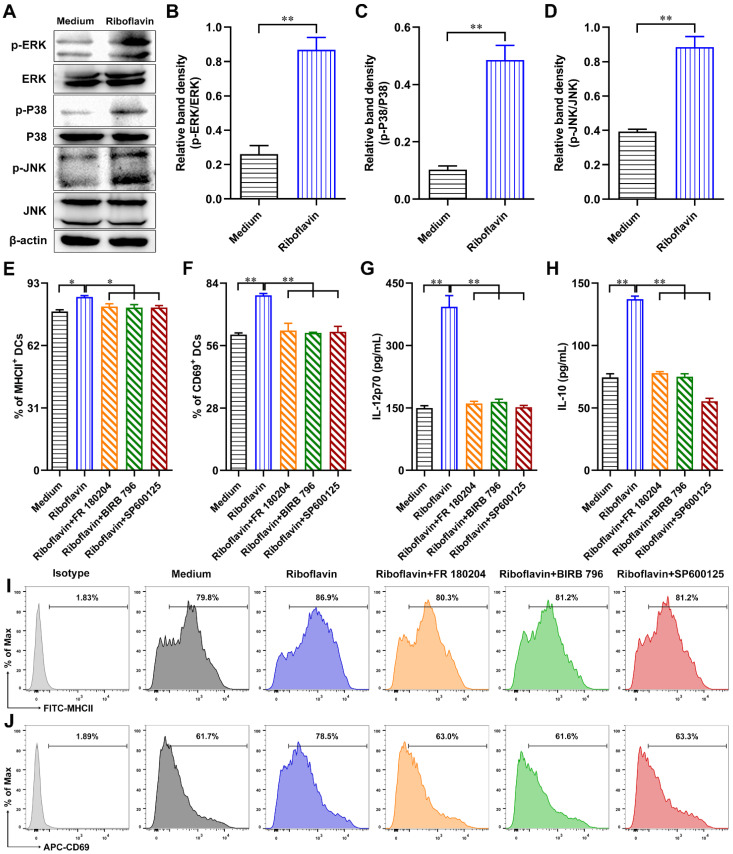
Riboflavin promotes DC maturation via the MAPK pathway. (**A**–**D**) DCs were incubated with riboflavin (40 µM) for 1 h, and then the expressions of p-ERK, ERK, p-P38, P38, p-JNK, JNK, and β-actin were detected by Western blot. (**E**–**J**) DCs were incubated with FR 180204 (ERK inhibitor, 10 µM), BIRB 796 (P38 MAPK inhibitor, 10 µM), or SP600125 (JNK inhibitor, 10 µM) for 2 h, followed by riboflavin for 24 h. The expressions of MHCII (**E**,**I**) and CD69 (**F**,J) in DCs were detected via FCM, and then the secretions of IL-12p70 (**G**) and IL-10 (**H**) in the supernatant were detected by ELISA. Results are from one representative experiment out of three performed. All of the data are presented as the mean ± SD. Statistical significance was assessed by unpaired Student’s two-sided *t*-tests or one-way ANOVA analysis to compare the results between the two groups or different groups, respectively; * *p* < 0.05, ** *p* < 0.01.

To demonstrate whether riboflavin might modulate DC maturation via the mitogen-activated protein kinase (MAPK) signaling pathway, DCs were incubated with riboflavin for 1 h, and then the expression levels of p-ERK, ERK, p-P38, P38, p-JNK, JNK, and β-actin were detected by Western blot. As shown in Figure 8A, the expressions of ERK, P38, and JNK in riboflavin-induced DCs showed no change compared with the medium control, whereas the expressions of p-ERK, p-P38, and p-JNK were highly upregulated compared with the medium control (Figure 8A–D). We further investigated whether the MAPK signaling pathway played a vital role in the DC maturation induced by riboflavin. The phenotypic marker (MHCII) (Figure 8E,I), activation marker (CD69) (Figure 8F,J), and cytokine release (IL-12p70 and IL-10) (Figure 8G,H) were detected when MAPK inhibitors were used. The results showed that the DC maturation induced by riboflavin was diminished when DCs were pretreated with FR 180204 (ERK inhibitor), BIRB 796 (P38 MAPK inhibitor), or SP600125 (JNK inhibitor) (Figure 8E–J). Therefore, the MAPK signaling pathway plays a remarkable role in the modulation of DC maturation by riboflavin.

## 4. Discussion

**Figure 9:**
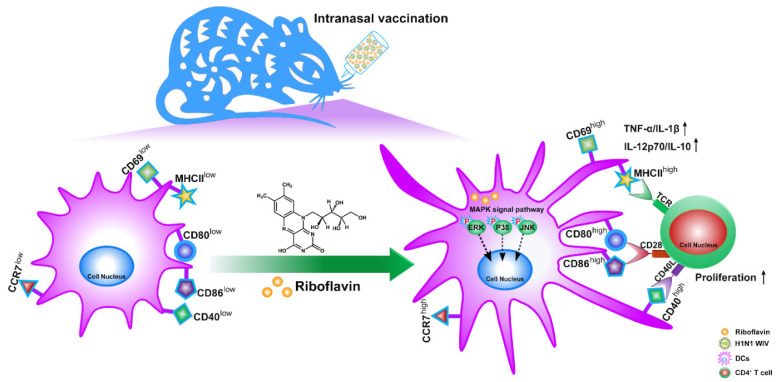
Schematic of the proposed mechanism by which riboflavin improves the immune response. Riboflavin first activated the MAPK signaling pathway, and then induced DC maturation, including through an activation marker (CD69), cytokine secretion (TNF-α, IL-1β, IL-12p70, and IL-10), phenotypic markers (MHCII, CD40, CD80, and CD86), a migration marker (CCR7), and the proliferation of allogeneic T cells. Furthermore, riboflavin enabled inactivated influenza nasal vaccine to trigger the downstream mucosal and systemic immune responses.

Our work reports that riboflavin, as a safe mucosal adjuvant, strongly increased the local mucosal and systemic immune response of inactivated influenza nasal vaccine in mice and further improved the protection levels from the lethal attack of H1N1 influenza virus (Figure 9). Moreover, we found that riboflavin possessed a strong capacity to induce DC maturation, including through phenotypic maturation, CD69 activation, the high expression of cytokines, the upregulation of migration marker CCR7, and the allostimulatory capacity of CD4^+^ T cells, which is a critical process for triggering downstream immune responses. Lastly, it was found that the DC maturation induced by riboflavin was dependent on the activation of the MAPK pathway (Figure 9).

The toxicity of CT is worrisome; therefore, attenuated CTB was developed [24], but its relatively high price limits its widespread use. Hence, the biosafety and price need to be considered during the development of mucosal adjuvants. Riboflavin, a nutrient necessary for maintaining good health in humans and animals, has been widely applied in the food industry as a safe food additive. To date, no complications associated with riboflavin supplementation have been reported, even when provided at very high doses, because excess riboflavin is excreted from urine as riboflavin or riboflavin-derived metabolites [12]. We also demonstrated its safety in mice by giving different doses of riboflavin intranasally (5 mg/kg, 10 mg/kg, and 20 mg/kg), which confirmed the good biosafety of riboflavin when applied in nasal inoculation. Riboflavin has been traditionally synthesized for food and feed fortification by chemical means. However, biotechnological processes with a low price based on bacteria, yeast, and fungi have shown strong commercial competition and have recently begun to replace chemical synthesis [25]. Here, considering its advantages of biosafety and low cost, we further explored whether riboflavin could be a potent mucosal adjuvant to improve the mucosal and systemic immune response of inactivated influenza nasal vaccine. Mucosal immunization has the advantage that specific sIgA antibodies are produced to neutralize viruses on the mucosal surface in the preliminary stage when the virus invades the host [26]. We found that the mice intranasally inoculated with H1N1 WIV plus riboflavin produced a higher level of specific sIgA in nasal, tracheal, and lung wash compared with H1N1 WIV alone, implying that riboflavin processed the ability to enhance the level of respiratory mucosal immunity. It is well known that the levels of IgG1 and IgG2a reflect the skewing of humoral and cellular immunity, respectively [27]. Our data showed that the IgG1/IgG2a ratio in the riboflavin group was significantly decreased compared with that in the group of H1N1 WIV alone, implying that riboflavin helped to strengthen the levels of cellular immunity, which is a key process for the elimination of intracellular infected viruses [28]. According to this strong adaptive immunity, riboflavin led to our H1N1 WIV nasal vaccine with one-third of the antigen dosage offering a 90% protection against fatal influenza virus, which is comparable with CTB [5,8,18].

DCs are vital for the initiation of T-cell immunity; in addition, they help to determine the course of the subsequent immune response [29]. Therefore, we next studied whether riboflavin could activate DCs in vitro. Four phenotypes that are critical for DC maturation, namely, MHC Ⅱ, CD40, CD80, and CD86 [30,31,32]. Our results found that these maturation phenotypes of DCs were upregulated after incubation with riboflavin, indicating that riboflavin enhanced the phenotypic maturation of DCs. DCs stimulated on the functional level exhibit the ability to secrete cytokines such as TNF-α, IL-1β, IL-12p70, and IL-10 [33]. Interestingly, our results showed that the levels of TNF-α, IL-1β, IL-12p70, and IL-10 were also markedly increased in the supernatant of the riboflavin group compared with the control, implying that riboflavin activated the functional maturation of DCs. We also found that the migration phenotype CCR7 of DCs was upregulated after the same treatment, which is beneficial to DC migration toward draining lymph nodes for antigen presentation [34]. After migration, complete DC maturation is vital for the activation and differentiation of naïve T cells [33,35]. Our study demonstrated that DCs incubated with riboflavin remarkably promoted the proliferation of allogeneic CD4^+^ T lymphocytes compared with antigen alone, hinting that riboflavin promoted complete DC maturation, triggering the proliferation and differentiation of downstream T cells. The epithelium of the nasal mucosa forms a continuous barrier against a wide variety of allothigenes [36]. DCs located beneath the epithelium can uptake influenza WIV across the mucosal epithelial barriers and present antigens to lymphocytes [8,37]. Therefore, the question of how to cross the epithelial barrier is also a consideration for mucosal adjuvant. A previous report found that riboflavin can pass through epithelial cells using riboflavin transporters [38], which led to us speculating that riboflavin could easily cross the epithelial barriers to activate DCs for the transepithelial uptake of influenza WIV and then trigger DC maturation and the downstream immune response. According to the above analysis, we believe that riboflavin is a promising mucosal adjuvant candidate for the application of nasal influenza vaccines.

MAPK cascades are key signaling pathways that regulate a wide variety of cellular processes, including immune response, inflammation, differentiation, proliferation, apoptosis, and stress responses [39]. P38, JNK, and ERK are key nodes in the MAPK signaling pathway [40,41]. Our work has proven that riboflavin upregulates the expression of p-P38, p-JNK, and p-ERK in DCs. Furthermore, MHC Ⅱ, CD69, IL-12p70, and IL-10 were successfully inhibited when P38 (BIRB 796), JNK (SP600125), and ERK (FR 180204) inhibitors were used. These data suggest that the DC maturation activated by riboflavin was dependent on the MAPK signaling pathway, which might be an underlying mechanism for explaining the immune activation characteristic of riboflavin.

## 5. Conclusions

In conclusion, we present a novel functional property of riboflavin based on the activation of DC maturation via the MAPK signaling pathway, helping the inactivated influenza nasal vaccine to remarkably improve the mucosal and systemic immune response and providing strong protection from the lethal attack of influenza virus. Therefore, riboflavin is likely to be developed as a potent mucosal adjuvant for mucosal vaccine application.

## Data Availability

The data presented in this study are available upon request from the corresponding author.

## Supplementary Materials

The following are available online at https://www.mdpi.com/article/10.3390/vaccines9111296/s1: Figure S1. The proliferation of splenic lymphocytes by riboflavin in vitro.
