# Supplementary material for: Riboflavin as a Mucosal Adjuvant for Nasal Influenza Vaccine
Source: Vaccines (Basel). 2021 Nov 9;9(11):1296. doi: 10.3390/vaccines9111296 (PMC8617635; doi:10.3390/vaccines9111296)
Supplement: Supplementary file 1 [file vaccines-09-01296-s001.zip › vaccines-1435942-SI.pdf]

vaccines

MDPI

Article

Riboflavin as a Mucosal Adjuvant for Nasal Influenza Vaccine

Yinyan Yin [1,†], Jinyuan Wang [2,3,4,5,†], Xing Xu [2,3,4,5], Bangyue Zhou [1,6], Sujuan Chen [2,3,4,5], Tao Qin [2,3,4,5], Daxin Peng [2,3,4,5,*]

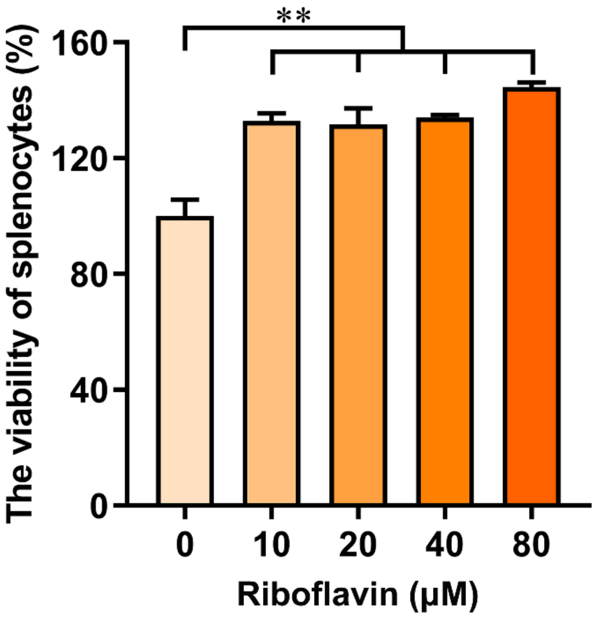

Figure S1. The proliferation of splenic lymphocytes by riboflavin in vitro. Lymphocytes were separated from spleens of BALB/c mice and incubated with riboflavin (10, 20, 40, and 80 μM) for 72 h, and then the proliferative level was detected by using the CCK-8 assay. Results are from one representative experiment of three performed. All of the data are presented as mean±s.d. Statistical significance was assessed by one-way ANOVA analysis to compare the results between different groups. ** $p < 0.01$.
